# Structures and Solvation Energies Effects Versus Temperature. An MP2 Investigations in the Framework of Cluster Model

**DOI:** 10.1002/jcc.70066

**Published:** 2025-02-19

**Authors:** Awatef Hattab, Alhadji Malloum, Zoubeida Dhaouadi, Nino Russo

**Affiliations:** ^1^ Laboratoire de Spectroscopie Atomique Moléculaire et Applications, Faculté des Sciences de Tunis, Université de Tunis El Manar Campus Universitaire Tunis Tunisie; ^2^ Faculté des Sciences de Bizerte Université de Carthage Zarzouna Bizerte Tunisie; ^3^ Department of Physics, Faculty of Science The University of Ngaoundere Ngaoundere Cameroon; ^4^ Dipartimento di Chimica e Tecnologie Chimiche Universitá della Calabria Rende (CS) Italy

**Keywords:** beryllium solvation, cluster structures, MP2, solvation energies, temperature dependence

## Abstract

Structures, relative stabilities, solvation enthalpies, and free energies of the $\;{\left[\mathrm{Be}{\left(H_{2}O\right)}_{n=12}\right]}^{2+}$ cluster in gas and in water phases were investigated in this work using Moller‐Plesset perturbation theory (MP2) and considering a temperature range of 40–400 K. The 12 H_2_O molecules are distributed between the first, second, and third solvation shells. The calculated distances $\;{\mathrm{Be}}^{2+}-O$ distances in gas phase are in good agreement with the experimental range which confirms the strong influence of long‐distance interactions in cluster stabilization. Structural comparison between gas and water phases shows that the addition of the bulk solvent causes changes in the cation‐water bond lengths of few hundredths of angstroms. The obtained solvation free energy of beryllium ion in water at room temperature (298.15 K) results in *b* − 575.1 kcal mol^−1^ in very good agreement with the corresponding experimental counterpart. The computed solvation free energies increase as a polynomial function of the temperature while the change in the solvation enthalpies is found to be negligible.

## Graphical abstract

The geometrical and energetic properties of the [Mg(NH_3_)_*n*=1–10_]^+^ clusters have been investigated at the MP2 level of theory. The isomeric distribution of the four stable structures has been studied as a function of temperatures (25–400 K).

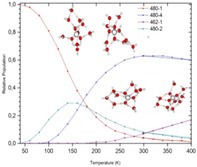

## 1 Introduction

The thermodynamic and spectroscopic properties of electrolyte solutions [1, 2, 3, 4, 5, 6, 7, 8, 9, 10] can depend from the distribution of solvent molecules around a given ion. In fact, hydration behavior of charged alkaline earth ion, including the beryllium cation ${\mathrm{Be}}^{2+}$, has attracted many experimental [1, 2, 5, 8, 11, 12, 13, 14, 15, 16, 17] and theoretical [3, 6, 7, 18, 19, 20, 21, 22, 23, 24, 25, 26, 27, 28, 29] attention in the recent years. The topology of water molecules around the $\;{\mathrm{Be}}^{2+}$ cation has been computationally determined for the first and second coordination shell [3, 4, 6, 7], by using up to 12 water molecules [4, 25] distributed between the first and second coordination shells.

In our previous MP2 study on the solvation of ${\mathrm{Be}}^{2+}$ cation in NH_3_, CH_3_OH, and H_2_O [27], we found that in the case of ${\left[\mathrm{Be}{\left(H_{2}O\right)}_{n}\right]}^{2+}$ cluster, the second shell can hold up to eight water molecules (*n* = 12). When the second solvation shell is saturated, we found that the ${\mathrm{Be}}^{2+}-O$ bond lengths match well with experimental results.

These finding corroborate the earlier proposal of Markham et al. [3] and Pye et al. [25] about the importance of adding molecules in the second solvation shell and suggest the ${\left[\mathrm{Be}{\left(H_{2}O\right)}_{12}\right]}^{2+}$ cluster as a reliable model.

For this reasons, we decided to use the $\;{\left[\mathrm{Be}{\left(H_{2}O\right)}_{n=12}\right]}^{2+}$ cluster in both the gas and in aqueous environments.

The solvation energies of ${\left[\mathrm{Be}{\left(H_{2}O\right)}_{4}\right]}^{2+}$ complex at *T* = 298 K has been determined by Markham et al. [3] using MP2 level of theory obtaining a $∆G_{298}$ and $∆H_{298}$ values of 352 and 363 kcal/mol, respectively. The presence of two water molecules in the second solvation shell increases the Gibbs free energy value ($∆G_{298}=395\;\text{kcal}/\mathrm{mol}$) and further addition of solvent molecules bring it to 421 kcal/mol [3, 4]. This value is in good agreement with the experimental one (575 kcal/mol) [1, 2] showing the important role of long‐range interactions in the stability of beryllium‐water clusters.

Despite the inclusion of a large number of water molecules in the second solvation shell being expensive by using MP2 level of theory, it is suggested as the most appropriate method to study these systems and to obtain accurate thermodynamic information [27]. In particular, the long‐range interactions, important in the cluster stabilization, are correctly reproduced, as shown in the work of Gnanakaran et al. [28].

Up to now, no works were devoted to investigate the effect of temperature on the relative population of ${\left[\mathrm{Be}{\left(H_{2}O\right)}_{12}\right]}^{2+}$ clusters, nor the solvent effects on the predicted isomers of the considered complex. As a result, there are no solvation energies of the ${\left[\mathrm{Be}{\left(H_{2}O\right)}_{n}\right]}^{2+}$ at temperatures other than 298 K.

In this work, we report the result of a computational investigation, performed at the MP2 level of theory, in which the solvent effects on ${\left[\mathrm{Be}{\left(H_{2}O\right)}_{12}\right]}^{2+}$ cluster, was simulated using the polarizable continuum model (PCM). The temperature effects on the isomeric stability and solvation energies (free energies and enthalpies) were carried out in the wide temperature range from *T* = 40–400 K.

We underline that this study cannot describe the interconversion between different structures, which is likely possible and may affect the thermodynamic properties. However, it can provide significant information and help to develop the force field for consecutive dynamical studies, which require very high computational resources.

## 2 Methodology

Gaussian 09 [30] code was used for geometry optimizations and frequency determination. The geometry and frequency calculations have been performed using MP2/6‐311++G (d,p) level of theory. The thermodynamic parameters have been evaluated at temperature of 298.15 K and under pressure of 1 atm. For all the calculations, the following default convergence criteria were used: root mean square force = 1.10^−5^, root mean square displacement = 4.10^−5^, maximum force = 1.5.10^−5^, and maximum displacement = 6.10^−5^.

Frequency calculations were performed to ensure that the optimized structures are local minima on the potential energy surfaces (PESs) of the considered clusters.

A revised version of Tempo [31] program was used to obtain the temperature effects on the relative population of the isomers and the free energies and enthalpies in the 40 to 400 K temperature range.

The change in the stability of isomers as a function of temperature was obtained from their canonical probabilities defined as:

(1) $$P_{n}^{\left(k\right)}\left(T\right)=\frac{\exp^{{-\mathrm{\beta G}}_{n}^{\left(k\right)}\left(T\right)}}{\sum_{k}\exp^{{-\mathrm{\beta G}}_{n}^{\left(k\right)}\left(T\right)}}$$

where $\beta =1/k_{B}T$, $k_{B}$ is the Boltzmann constant, $G_{n}^{\left(k\right)}\left(T\right)$ is the free energy of the kth isomer of size n at the temperature *T*, as calculated by the Tempo code.

From previous thermodynamics work of some particles in the gas phase [32, 33], it was found that the probability distribution of these complexes could be strongly influenced by quantum effects at cryogenic temperatures, especially for *T* < 20 K. Therefore, the temperature‐dependent relative stability is discussed here for *T* > 20 K.

The isomers were defined by *ijk* symbols that represent the *i*, *j*, and *k* number of water molecules in the first, second, and third coordination shells, respectively. The sum of *i* + *j* + *k* is equal to *n*. When *m* isomers are found in the same *ijk* configuration, the new label *ijk*‐*m* is used.

**FIGURE 1:**
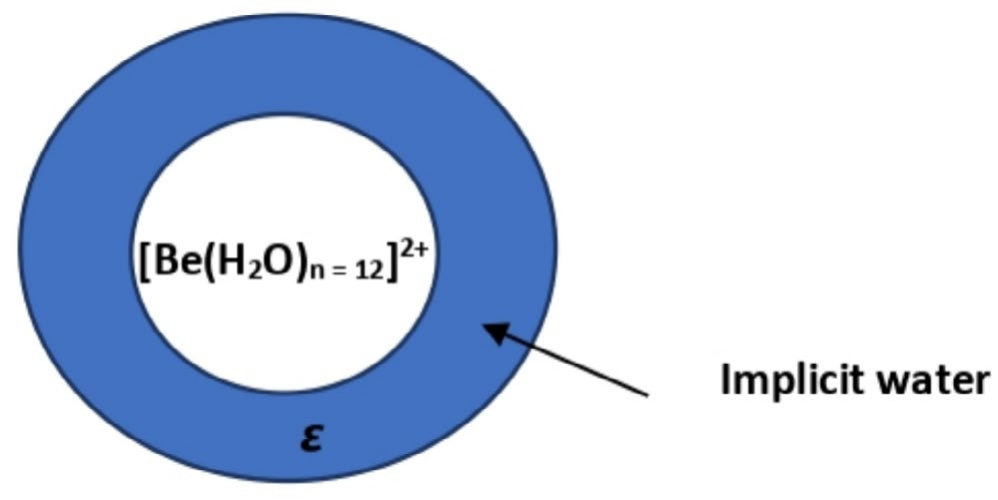
Scheme of the hydration of the beryllium cation. The blue sphere represents the bulk aqueous solvent described as a polar dielectric continuum medium.

Since the explicit treatment of a great number of water molecules is unrealistic, a high ab initio level of mixed solvation process was used. In this model, the cluster was treated explicitly and the solvent bulk effects were treated as a polar dielectric continuum medium (Figure 1) in the framework of the Integral Equation Formalism Polarized Continuum Model (IEFPCM) [34]. The value of = 78.3553 was used for the dielectric constant of water.

The solvation energies ($∆G_{s}$) and ($∆H_{s}$) of the beryllium ion in water at a specific temperature for the process

(2) $${\mathrm{Be}}^{2+}\;\left(\mathrm{gas}\right)+{\left(H_{2}O\right)}_{n=12}\;\left(\text{water}\right)\;{\mathrm{Be}}^{2+}{\left(H_{2}O\right)}_{n}\;\left(\text{water}\right)$$

were calculated by using the following equations:

(3) $$∆G_{s}{\left({\mathrm{Be}}^{2+}\right)}_{n}=∆G_{s}\left[{\mathrm{Be}}^{2+}{\left(H_{2}O\right)}_{n}\right]-∆G_{s}\left[{\left(H_{2}O\right)}_{n}\right]-∆G_{\mathrm{gas}}\left({\mathrm{Be}}^{2+}\right)$$

(4) $$∆H_{s}{\left({\mathrm{Be}}^{2+}\right)}_{n}=∆H_{s}\left[{\mathrm{Be}}^{2+}{\left(H_{2}O\right)}_{n}\right]-∆H_{s}\left[{\left(H_{2}O\right)}_{n}\right]-∆H_{\mathrm{gas}}\left({\mathrm{Be}}^{2+}\right)$$

in which the first two terms noted $\begin{array}{c} ∆G_{s}\left[{\mathrm{Be}}^{2+}{\left(H_{2}O\right)}_{n}\right]/ \end{array}\begin{array}{c} ∆H_{s}\left[{\mathrm{Be}}^{2+}{\left(H_{2}O\right)}_{n}\right] \end{array}$ and $∆G_{s}\left[{\left(H_{2}O\right)}_{n}\right]/∆H_{s}\left[{\left(H_{2}O\right)}_{n}\right]$ stand for solvation energies of beryllium‐solvent cluster and water molecule cluster ${\left(H_{2}O\right)}_{n}$, respectively. These terms are calculated using the IEFPCM model. $∆G_{\mathrm{gas}}\left({\mathrm{Be}}^{2+}\right)$ and $∆H_{\mathrm{gas}}\left({\mathrm{Be}}^{2+}\right)$ are respectively, the free energy and the enthalpy of the Beryllium cation in gas phase.

It is important to note that the thermodynamic properties are calculated based on rigid‐rotor and harmonic approximations. Furthermore, the intermolecular interactions between the molecules are considered negligible. These approximations can affect the accuracy of the calculated thermodynamic properties. Nevertheless, regarding the hydration enthalpy and free energy of the beryllium ion, some error cancellations are expected to occur based on Equations (3) and (4). Therefore, these error cancellations considerably reduce the overall error on the hydration enthalpy and free energy of the beryllium ion.

## 3 Results and Discussion

### 3.1 Gas Phase Results

**FIGURE 2:**
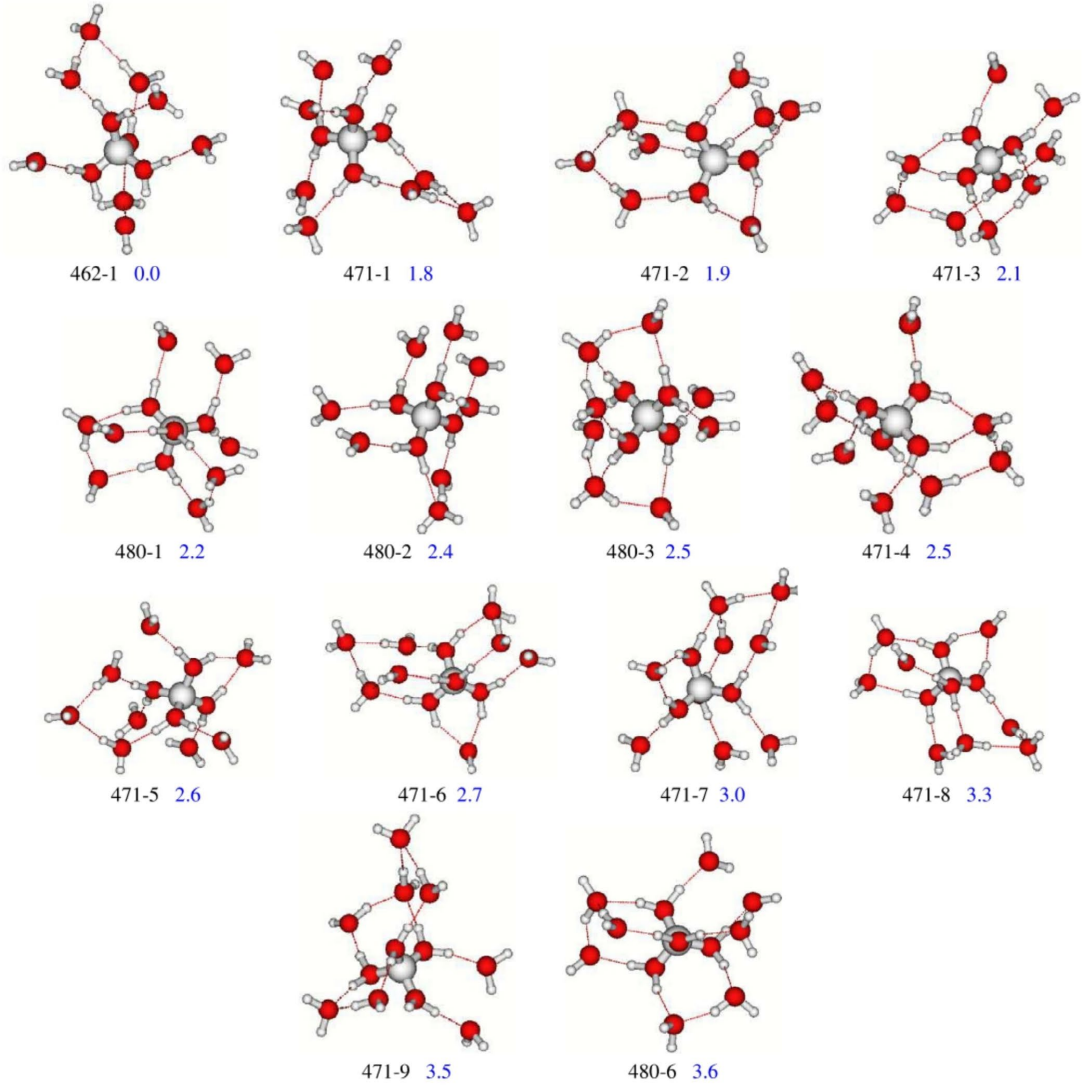
Structures of the most stable isomers of $\;{\left[\mathrm{Be}{\left(H_{2}O\right)}_{n=12}\right]}^{2+}$ complex in gas phase using MP2/6–311++G(d,p) computational level. Cluster definitions are reported on Section 2. Numbers represent the zero‐point‐corrected electronic energy differences in kcal/mol (blue color).

Different structures were characterized for the $\;{\left[\mathrm{Be}{\left(H_{2}O\right)}_{n=12}\right]}^{2+}$ cluster (Figure 2). In the most stable isomer (462–1) two water molecules are located in the third hydration shells with a ${\mathrm{Be}}^{2+}-O$ bond length of 1.634Å and a valence angle H—O—H of111.3°(see Table 1). These calculated values are in agreement with the experimental range [3, 14, 16, 38, 39].

**TABLE 1:** Optimized geometrical bond lengths (Å) and angles (degree) for the first solvation shell for the three most stable isomers of $\;{\left[\mathrm{Be}{\left(H_{2}O\right)}_{n=12}\right]}^{2+}$ complex in the gas phase and in aqueous solution.

Structures (*ijk*)	$d_{{\mathrm{Be}}^{2+}-O}$			$d_{O—H}$			∠H—O—H		
	Gas phase	Solv	Exp [5, 8, 11, 12, 13, 14, 15, 17, 35, 36]	Gas phase	Solv	Exp [8, 17]	Gas phase	Solv	Exp [17]
480–1	1.635	1.640	1.61–1.69	0.986	0.988	0.967–0.971	109.6	108.9	112.7
471–1	1.635	1.640		0.985	0.987		110.5	109.2	
462–1	1.634	1.635		0.987	0.987		111.3	111.0	
$H_{2}O$				0.958 [37]			104.5 [37]		

Furthermore, we characterize three isomers, 471‐1, 471‐2, and 471‐3, which are quite degenerated in energy, lying at 1.8, 1.9, and 2.1 kcal/mol above the absolute one, respectively (Figure 2).

The ${\mathrm{Be}}^{2+}-O$ bond length of 471‐1 is found to be 1.635Å and the H—O—H angle is 110.5° (see Table 1).

The less stable isomers 480‐1, 480‐2, and 480‐3 are also almost degenerate and lying at 2.2, 2.4, and 2.5 kcal/mol above the most stable one. The lower stability of these isomers can be attributed to the absence of water molecules in the third solvation shell. The ${\mathrm{Be}}^{2+}-O$ distances in the 480‐1 $\left(1.635\;Å\right)$ is slightly longer compared to that of the global minimum 462‐1 $\left(1.634\;Å\right)$.

The energetic differences between all these isomers can be explained by the HB‐types, which A stands for acceptor and D for donor. In fact, A‐type hydrogen bonds (HBs) act as stabilizer, however AA and AAD‐type HB act as destabilizers, as also reported [35].

We underline that the addition of solvent molecules in the third coordination sphere leads to the shortening of the cation‐oxygen bond length and allows to converge more and more towards the experimental data [5], and enforce the important role of long‐range interactions in the cluster stabilization as we have already reported in our previous study [27].

Six isomers 471‐4, 471‐5, 471‐6, 471‐7, 471‐8, and 471‐9 have also been characterized at 2.5, 2.6, 2.7, 3.0, 3.3, and 3.5 kcal/mol above the most stable one and are less stable than that with a non‐occupied third solvation shell (480‐1‐480‐3).

The multi bridging structure 480‐6 (3.6 kcal/mol), which is built by a double acceptor (AA‐type), acceptor‐donor (AD‐type), and double acceptor‐donor (AAD‐type) is less stable than the 480‐3 (2.5 kcal/mol), suggesting that this minor stability can be related to the HB network's in ${\left[\mathrm{Be}{\left(H_{2}O\right)}_{n=12}\right]}^{2+}$ clusters.

On the basis of these results, we suggest that both long‐range interactions and hydrogen‐bond types influence the stability of isomers (with the same *ijk* configuration) of the Beryllium‐water complex.

**FIGURE 3:**
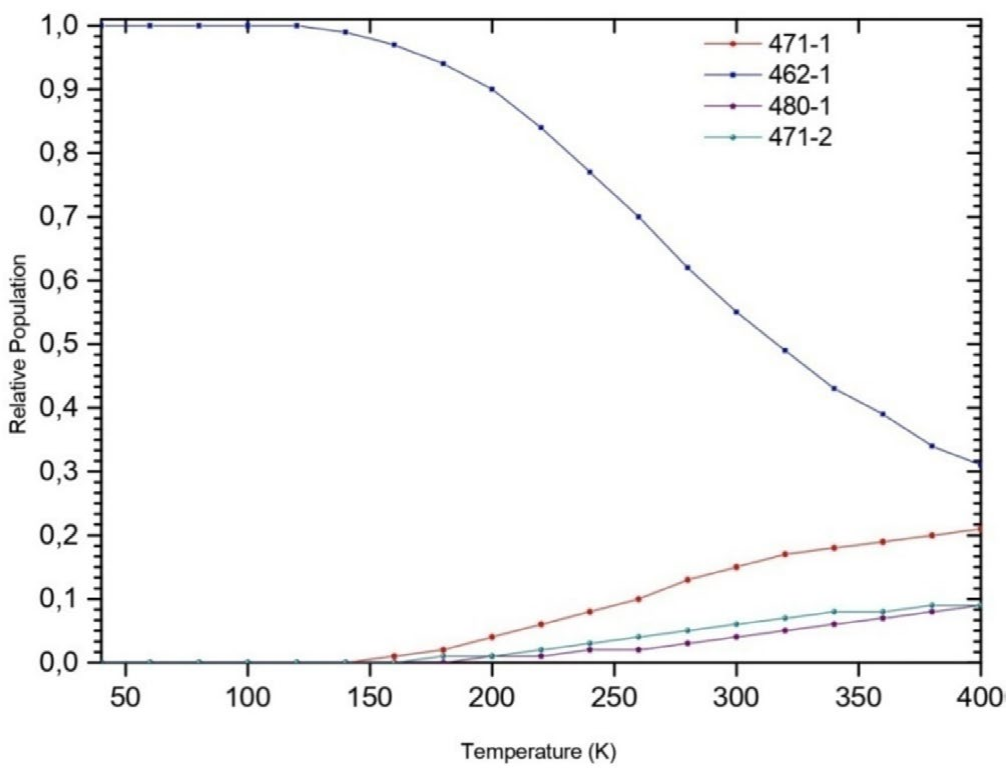
Temperature‐dependent of the relative populations of $\;{\left[\mathrm{Be}{\left(H_{2}O\right)}_{n=12}\right]}^{2+}$ isomers in gas phase.

The change of the stability of isomers as a function of temperature shows that the 462‐1 isomer strongly dominates at low temperatures, while at *T* = 280 K, the 471‐1 form contributes 13%. As the temperature increases, the 462‐1 population slightly decreases, however that of 471‐1 follows an opposite behavior, as shown in Figure 3. A slight contribution (less than 10%) of the 471‐2 and 480‐1 isomers is noted at higher temperatures.

### 3.2 Solvated Phase Results

In simulated liquid‐phase, the most stable isomer of the $\;{\left[\mathrm{Be}{\left(H_{2}O\right)}_{n=12}\right]}^{2+}$ is the symmetric *C*_s_ structure 480‐1, characterized by a ${\mathrm{Be}}^{2+}-O$ bond length of 1.640Å and H—O—H valence angle of 108.9° (see Table 1).

**FIGURE 4:**
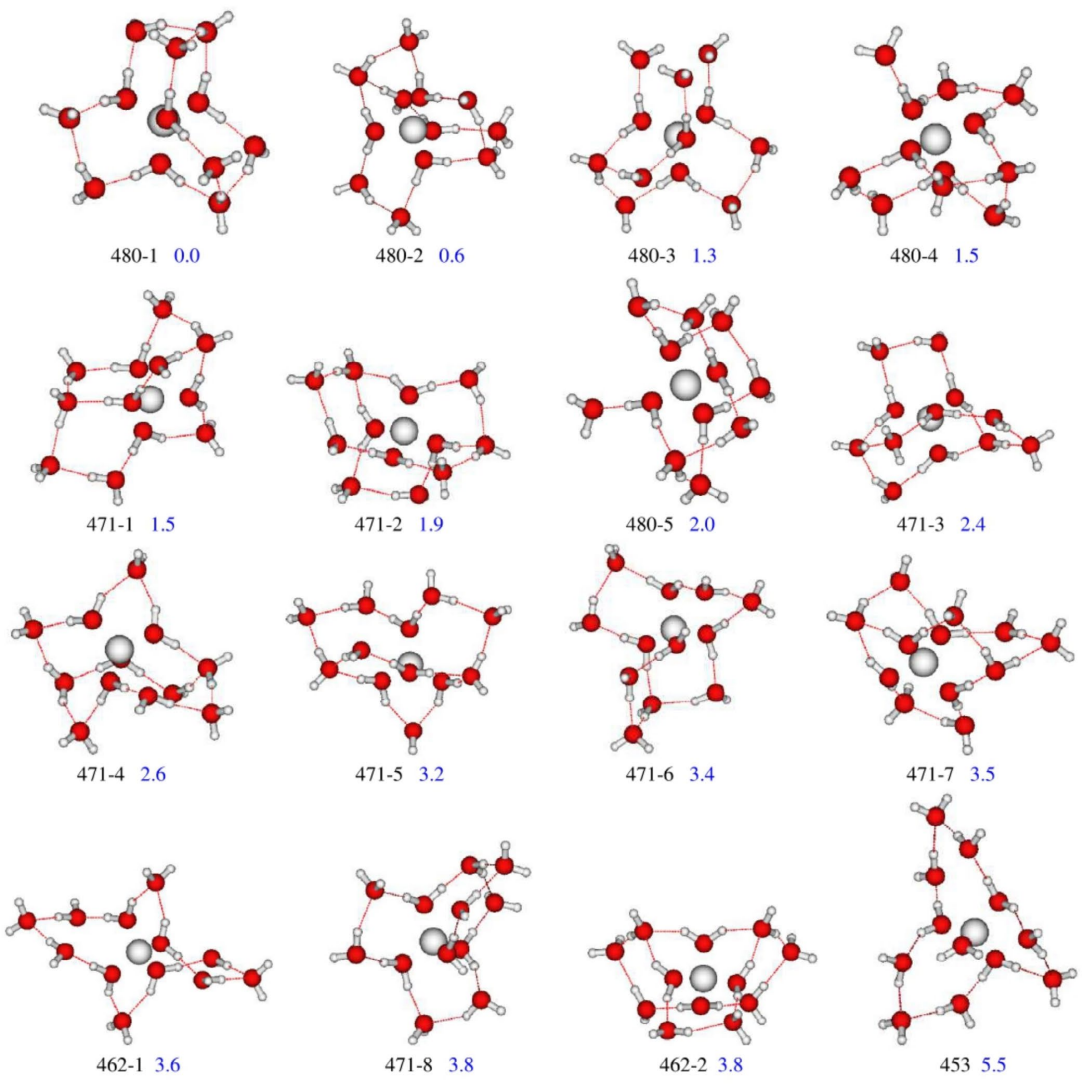
Structures of the most stable isomers of $\;{\left[\mathrm{Be}{\left(H_{2}O\right)}_{n=12}\right]}^{2+}$ complex in solvent phase using MP2/6–311++G(d,p) computational level. For the cluster definitions see the Methodology section. Numbers represent the zero‐point‐corrected electronic energy differences in kcal/mol (blue color).

Moreover, we have located four “branched structure” 480‐2, 480‐3, 480‐4, and 480‐5 on the PES lying at 0.6, 1.3, 1.5, and 2.0 kcal/mol above the most stable one (Figure 4).

The relative isomerization energy difference is attributed to the number of A‐type HB that acts as destabilizer. Other eight structures (471‐*m*) with 7 water ligands located in the second coordination shells and the rest in the third one were characterized as minima in the PES. These structures lie at 1.5, 1.9, 2.4, 2.6, 3.2, 3.4, 3.5, and 3.8 kcal/mol above the absolute minimum. Their energies difference may be ascribed to HB types in the cluster since AAD type‐HB acts as a stabilizer while A and AA types‐HB act as destabilizers.

Furthermore, two structures (462‐*m*), in which the second and the third spheres are occupied by 6 and 2 water molecules, respectively, are located at 3.6 and 3.8 kcal/mol above the energy of the 480‐1.

The energetic behavior between 471‐2 $\left(C_{2v}\right)$ and 480‐5, and between 462‐1 $\left(C_{2v}\right)$ and 471‐8 $\left(C_{1}\right)$ can be related to their symmetry since higher symmetry favors a higher stability [40, 41, 42, 43].

Finally, the intercepted structure with 5 and 3 water molecules in the second and the third solvation shell, respectively (453), is less stable and lies 5.5 kcal/mol above 480‐1.

We note that, contrary to gas phase results, the most stable complexes in solvent phase are those in which the second sphere is completely saturated (by eight water molecules). This can be interpreted by the fact that the closure of the second solvation sphere forms a metal cage around the cation, then the contribution of HB interactions becomes negligible with respect to the solvation phenomenon.

**FIGURE 5:**
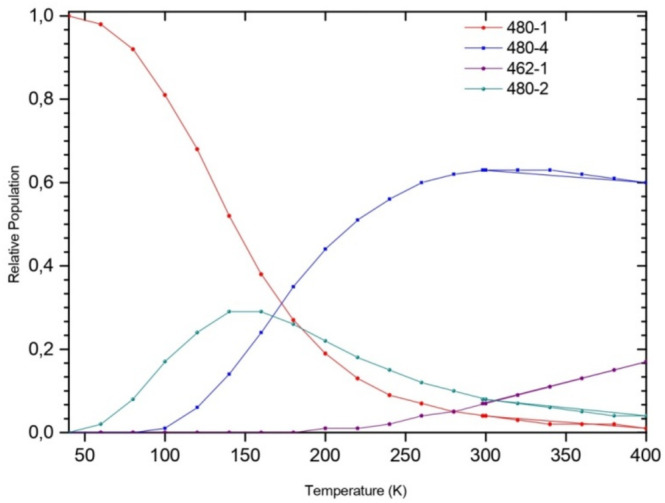
Temperature dependent of the relative populations of $\;{\left[\mathrm{Be}{\left(H_{2}O\right)}_{n=12}\right]}^{2+}$ isomers in water.

Our calculated distribution probabilities show that, from all possible structures located on the PES of the $\;{\left[\mathrm{Be}{\left(H_{2}O\right)}_{n=12}\right]}^{2+}$ complex (Figure 5), only 480‐1, 480‐2, 480‐4, and 462‐1 topologies could contribute to its population with more than 2%.

The behavior of the obtained temperature dependent population strongly indicates that at low temperature the fused multi‐cyclic structure, 480‐1 structure (Figure 5) is dominant. From ~80 K, also the 480‐2 isomer starts to contribute. However, for temperatures above 180 K, the population of the branched 480‐4 isomer and that of the fused multi‐cyclic structures 480‐1and 480‐2 decreases. At higher temperatures (*T* ≥ 260 K), 462‐1 isomer contributes with a probability ≤17%, while that of 480‐1 and 480‐2 result to be lower (≤10%).

### 3.3 How the Bulk Solvents Influence the Geometrical Structures of $\;{\left[\mathrm{Be}{\left(H_{2}O\right)}_{n=12}\right]}^{2+}$ Clusters?

The comparison of the main geometrical parameters of the 480‐1, 471‐1, and 462‐1 isomers in the considered environments (Table 1) with the available experimental show that in going from gas to water phase, the ${\mathrm{Be}}^{2+}-O$ bond lengths increase by ≈0.005 indicating as the presence of the bulk solvent does not have a significant effect. The same behavior was noted for the O−H distances (about 0.002 Å of increase).

On the contrary, the H—O—H valence angles decrease in a range of 0.3°–1.3°.

The slight difference between our calculated distances and the corresponding available experimental one (few hundredths of angstroms) can be explained by the fact that experimental measurements were performed using fairly concentrated solutions (> 1 M) while theoretical measurements refer to infinite dilution. This finding confirms the earlier suggestion of Sanchez Marcos et al. [4] about the influence of the concentration on the bond lengths.

### 3.4 Solvation Free Energies and Solvation Enthalpies Versus Temperature

**FIGURE 6:**
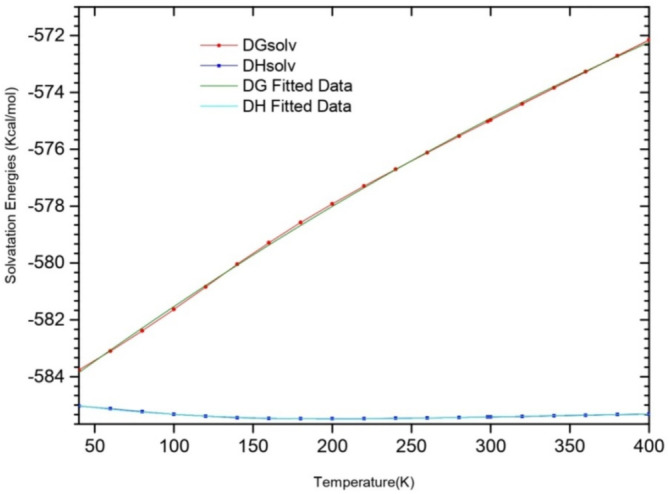
Solvation free energies versus solvation enthalpies of 480‐1 isomer at different temperatures.

In this section, we present how the temperature can influence the solvation enthalpies and solvation free energies of the Beryllium ion in water in the temperature range from 40 to 400 K (Figure 6). We underline that the solvation energies (enthalpy and free energy) were calculated here for the most stable isomer in solvent 480‐1 using PCM correction.

The solvation free energies $∆G_{\text{water}}{\left({\mathrm{Be}}^{2+}\right)}_{n}$ and enthalpies $∆H_{\text{water}}{\left({\mathrm{Be}}^{2+}\right)}_{n}$ of Beryllium ion in water increase as polynomial function of temperature: $∆{G,H}_{\text{water}}^{\mathrm{fit}}\left({\mathrm{Be}}^{2+},T\right)=a+a_{1}T+a_{2}T^{2}+a_{3}T^{3}$.

The corresponding correlation factor is = 0.9949 and the root mean square deviation between the fitted and the calculated solvation energies (enthalpy and free energy) is *R* ^2^=0.09067 kcal/mol. The values of the optimized parameters are as follows: *a* = −584.70854, $a_{1}$ = −0.00909, $a_{2}$ = 3.32214E−5, $a_{3}$ = −3.5888E−8. In fact, for this temperature range, $∆H_{\text{water}}{\left({\mathrm{Be}}^{2+}\right)}_{n}$ increases from −583.7 to −572.1 kcal/mol (by an average of 0.033 kcal.mol^−1^ per Kelvin) while $∆H_{\text{water}}{\left({\mathrm{Be}}^{2+}\right)}_{n}$ increases from −585.03 to −585.4 kcal/mol (by an average of 0.001 kcal.mol^−1^ per Kelvin).

As previously mentioned, the only available experimental values of the solvation free energy of the Beryllium in water ($∆G_{\text{water}}{\left({\mathrm{Be}}^{2+}\right)}_{n}$) is available at room temperature [1, 2] while the theoretical one, obtained at a lower level of theory employing a $\;{\left[\mathrm{Be}{\left(H_{2}O\right)}_{6}\right]}^{2+}$ [3, 4] cluster report a solvation free energies value of −421 kcal/mol in disagreement with the experimental one's (−575 kcal/mol).

It is worth noting that our calculated solvation free energy $∆G_{\text{water}}{\left({\mathrm{Be}}^{2+}\right)}_{n}$ at *T* = 298.15 K is −575.1 kcal/mol is in excellent agreement with the experimental counterpart. Thus, the use of a cluster with 12 water molecules explicitly and the use of the PCM is able to give reliable theoretical results comparable with the experimental determinations. Also this finding supports the previous suggestion about the importance of long‐range interaction in the determination of fundamental thermodynamic properties [3, 4, 23, 25, 27].

## 4 Conclusion

Structures, relative stabilities and solvation energies (free energy and enthalpy) of $\;{\left[\mathrm{Be}{\left(H_{2}O\right)}_{n=12}\right]}^{2+}$ isomersare reported here for the first time using MP2/6‐311++G(d,p) level of theory in both gas and water phases.

On the basis of this study the following conclusions can be drawn:

– Bulk effect induce a lengthens of the ${\mathrm{Be}}^{2+}-O$ bond by a few hundredths of angstroms.
– Contrary to gas phase, the most stable isomers in solvent phase are the 480‐*m*, in which the second sphere is completely saturated, suggesting that the closure of the second solvation shell forms a metal cage around the cation and that the contribution of HB interactions becomes negligible on the solvation phenomenon.
– The solvation free energy is found to be −575.1 kcal mol^−1^ in excellent agreement with that experimentally determined.
– The $\;{\left[\mathrm{Be}{\left(H_{2}O\right)}_{n=12}\right]}^{2+}$ complex seems to be a good model for an accurate theoretical description of the thermodynamic properties of Be^2+^ solvation process.

## Data Availability

The data that support the findings of this study are available on request from the corresponding author. The data are not publicly available due to privacy or ethical restrictions.
